# Successful treatment of drug reaction with eosinophilia and systemic symptoms secondary to multiple viral infections and nonsteroidal anti-inflammatory drugs in a patient with autoimmune hepatitis

**DOI:** 10.1097/MD.0000000000049839

**Published:** 2026-07-31

**Authors:** Shengwu Chuai, Yanhong Gao, Ran Wang, Qianqian Li, Xuyong Lin, Ting Wang, Xingshun Qi

**Affiliations:** a Department of Gastroenterology, General Hospital of Northern Theater Command (Teaching Hospital of Shenyang Pharmaceutical University), Shenyang, Liaoning, China; b Department of Pathology, First Affiliated Hospital of China Medical University, Shenyang, Liaoning, China; c Postgraduate College, Shenyang Pharmaceutical University, Shenyang, China.

**Keywords:** autoimmune hepatitis, cytomegalovirus, DRESS, glucocorticoids, NSAIDs

## Abstract

**Rationale:**

Drug reaction with eosinophilia and systemic symptoms (DRESS), a potentially life-threatening drug-induced adverse reaction, can damage various organs, but is under-recognized in clinical practice.

**Patient concerns:**

A 44-year-old female was admitted due to abnormal liver function for half a month. During hospitalization, she developed fever, rash, pleural effusion, and multiple organ dysfunction.

**Diagnoses:**

Probable DRESS was considered, except for cytomegalovirus, circovirus, Epstein–Barr virus, and herpes simplex virus infection as well as autoimmune hepatitis.

**Interventions:**

Antiviral and antibacterial infection drugs and nonsteroidal anti-inflammatory drugs were used, but fever was not improved. After the diagnosis of “probable DRESS,” methylprednisolone and immunoglobulin were added. Then, fever was controlled.

**Outcomes:**

Finally, her temperature normalized, and rashes around right quadrant abdomen and limbs gradually decreased. Lung computed tomography scan was repeated, indicating the improvement of pneumonia and pleural effusion. Serum bilirubin and immunoglobulin G levels also decreased.

**Lessons:**

DRESS should be considered in a patient suffering from viral infection and receiving potentially causative drugs, when fever, rash, and multiple organ dysfunction occur.

## 1. Introduction

Drug reaction with eosinophilia and systemic symptoms (DRESS) or drug-induced hypersensitivity syndrome is a rare and potentially life-threatening drug-induced adverse reaction with a mortality of up to 10%. Its main symptoms include fever, rash, and organ damage. However, in clinical practice, it is difficult to identify whether the potential cause of fever should be DRESS or viral infection. Additionally, rash often develops after 2 to 6 weeks of exposure to a drug, and there is no significant improvement in clinical symptoms after discontinuing the drug. A diagnosis of DRESS should be highly suspected, especially when severe rash combined with multiple organ dysfunction occur. The etiology of DRESS may be related to genetic susceptibility, abnormal immune responses, deficiencies in drug-metabolizing enzymes, and viral reactivation.^[1]^ Viral reactivation associated with DRESS mainly includes human herpes virus infections as well as cytomegalovirus, Epstein–Barr virus, and herpes simplex virus.^[2]^ The most common drugs for DRESS include allopurinol, sulfonamide drugs, aromatic antiepileptic drugs,^[3]^ antituberculosis drugs, vancomycin, antiretroviral agents,^[4]^ and nonsteroidal anti-inflammatory drugs (NSAIDs).^[5]^ The management of DRESS includes discontinuation of suspected allergenic drugs, protection of affected organs, and administration of systemic corticosteroids. Besides, intravenous immunoglobulin infusion may also be required in some cases.^[6]^ Herein, we report a female with autoimmune hepatitis (AIH) who developed fever, rash, and pleural effusion, and was finally diagnosed with “probable DRESS” secondary to viral infections and use of NSAIDs.

## 2. Case presentation

**Figure 1.**
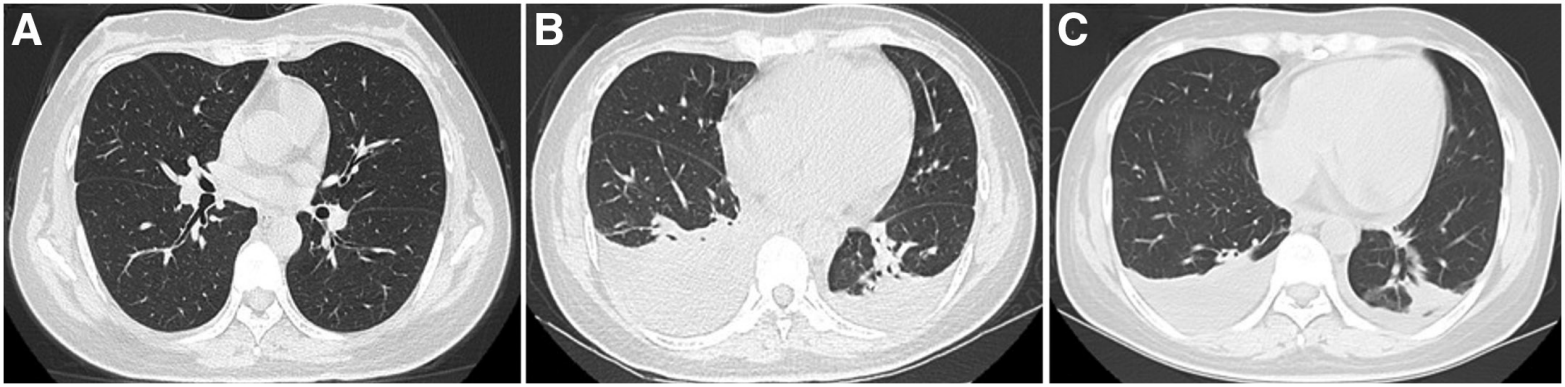
CT images of the lung. (A) lung CT scan on October 26, 2024 showing small nodules in both lungs without pleural effusion; (B) lung CT scan on November 5, 2024 showing pneumonia, bilateral pleural effusion, lymphadenopathy, as well as mild pericardial effusion; (C) lung CT scan on November 12, 2024 indicating the improvement of pneumonia and pleural effusion. CT = computed tomography.

**Figure 2.**
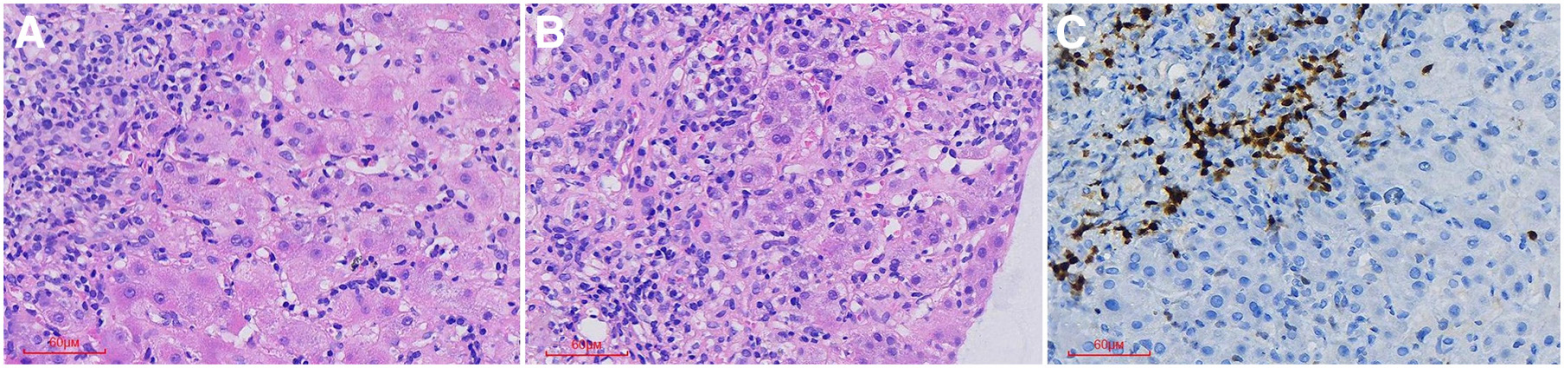
Liver pathological examination. (A) hematoxylin-eosin staining (200x) showing severe interfacial inflammation; (B) hematoxylin-eosin staining (200x) showing rose knot formation; (C) MUM1 staining showing severe interfacial inflammation with a large number of plasma cell infiltration. MUM1 = multiple myeloma oncogene 1.

**Figure 3.**
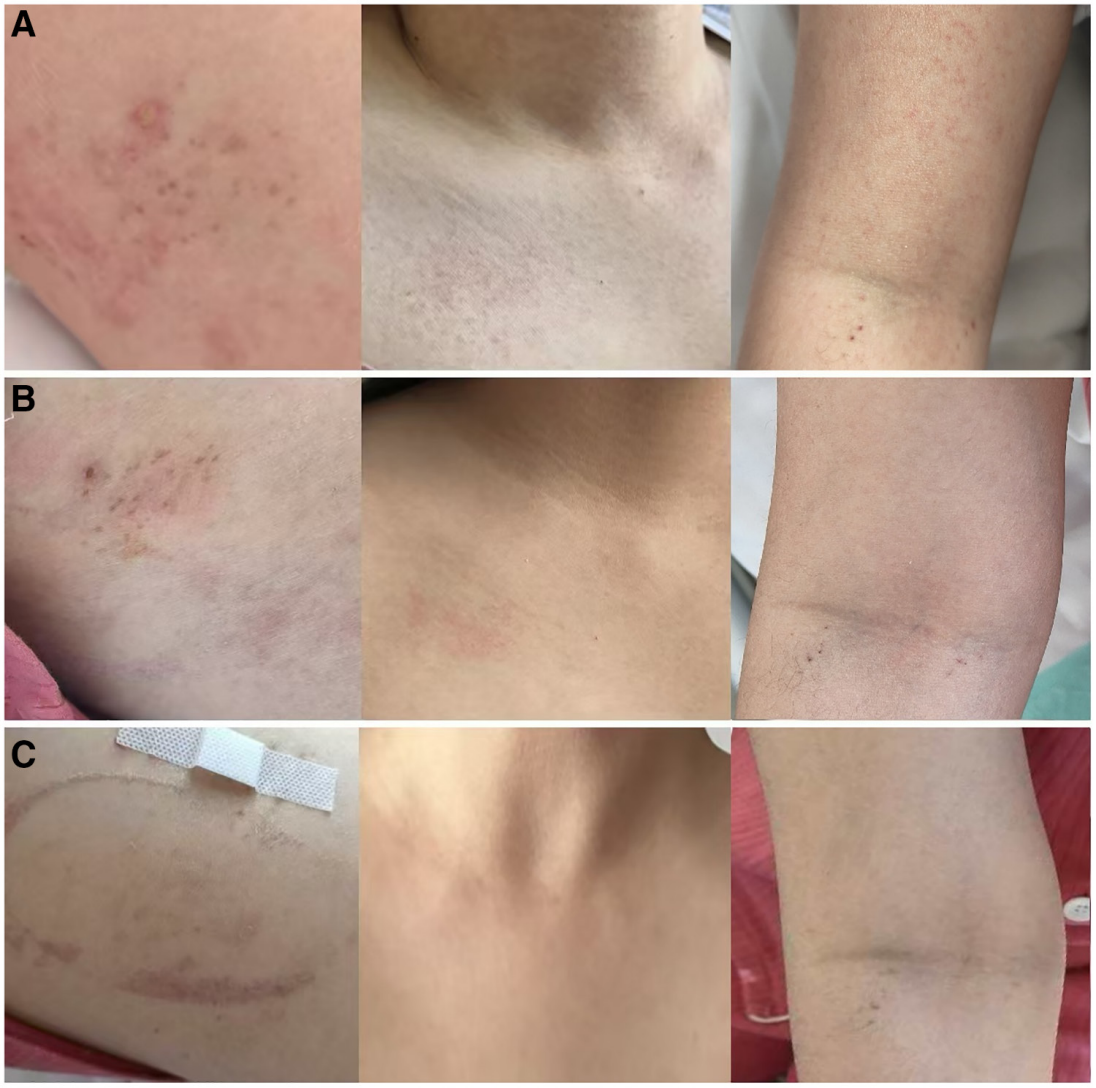
Change of rash. (A) occurrence of rash around the right upper abdominal, neck, prothorax, and arm on November 5; (B) decrease of rashes around the right quadrant abdomen, neck, prothorax, and arms on November 7; (C) complete resolution of rashes around the right upper abdominal, neck, prothorax, and arms on November 25.

**Figure 4.**
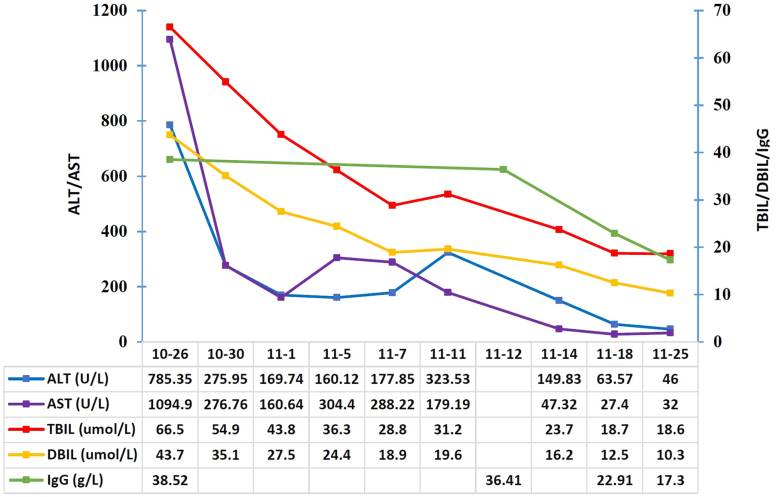
Change of transaminase, bilirubin, and IgG levels. ALT = serum alanine aminotransferase, AST = serum aspartate transaminase, DBIL = direct bilirubin, IgG = immunoglobulin G, TBIL = total bilirubin.

On October 25, 2024, a 44-year-old female was admitted to our department due to abnormal liver function for half a month. On October 4, she presented with fever with a peak body temperature of 38°C. She received ibuprofen, oseltamivir phosphate, and roxithromycin orally, and the fever resolved 1 week later. On October 11, the color Doppler ultrasound of the lymph nodes at local hospital showed that the lymph nodes in bilateral neck, supraclavicular fossa, and axillary fossa were enlarged. On October 12, Epstein–Barr virus panel demonstrated positive Epstein–Barr virus early antigen immunoglobulin (Ig)G antibody, strong positive Epstein–Barr virus capsid antigen IgG antibody, positive Epstein–Barr virus nuclear antigen IgG antibody, and negative Epstein–Barr virus capsid antigen IgM antibody. Routine blood test showed white blood cells 3.07 × 10^9^/L (reference range: 3.5–9.5 × 10^9^/L), hemoglobin 101 g/L (reference range: 115–150 g/L), eosinophils 0.00 × 10^9^/L (reference range: 0.02–0.52 × 10^9^/L), and lymphocytes 1.27 × 10^9^/L (reference range: 1.10–3.20 × 10^9^/L). At the same time, liver function tests demonstrated serum aspartate transaminase 1220 U/L (reference range: 13–35 U/L), serum alanine aminotransferase 894 U/L (reference range: 7–40 U/L), serum alkaline phosphatase 165 U/L (reference range: 35–100 U/L), serum gamma-glutamyltransferase 149 U/L (reference range: 7–45 U/L), and total bilirubin 56.5 μmol/L (reference range: 0.0–21.0 umol/L). Serological tests for hepatitis A, B, C, and E virus were negative. Abdominal color Doppler ultrasound indicated liver hemangioma and splenomegaly. She reported a 3-year history of recurrent fever and rash, but the cause was unclear. On October 26, plain magnetic resonance and magnetic resonance cholangiopancreatography scans demonstrated multiple liver lesions, hemangioma, cholecystitis, and splenomegaly. On October 26, 2024, computed tomography (CT) images of the lung demonstrated small nodules in both lungs (Fig. 1A). On October 26, 2024, cytomegalovirus infection was identified. On October 29, liver biopsy was performed. Before that, tranexamic acid and ketorolac were administered. Pathological examination demonstrated destruction of hepatic ductal areas, moderate to severe interface inflammation, lymphocyte infiltration, and severe bridging necrosis, suggesting a diagnosis of AIH (G4.S2-3) (Fig. 2). On the same day, after liver biopsy, she developed fever with a temperature of 38.7°C. On October 30, ganciclovir and indomethacin were given for antiviral and antipyretic treatment. Routine blood test showed white blood cells 2.3 × 10^9^/L (reference range:3.5–9.5 × 10^9^/L), eosinophils 0.00 × 10^9^/L (reference range: 0.02–0.52g × 10^9^/L), hemoglobin 85 g/L (reference range: 115–150 g/L), lymphocytes 0.85 × 10^9^/L (reference range: 1.10–3.20 × 10^9^/L), interleukin (IL)-6 10.79 pg/mL (reference range: 0–5.40 pg/mL), and IL-17 60.46 pg/mL (reference range:0–21.40 pg/mL). On November 4, cefoperazone and sulbactam were also given, but were replaced by meropenem due to the occurrence of rashes in the neck (Fig. 3A). Despite so, fever persisted, reaching a temperature of 39°C. Meanwhile, diphenhydramine and levocetirizine were prescribed for allergy. On November 5, her temperature rose to 39.5°C with dyspnea, facial edema, and low blood pressure of 89/52 mm Hg. Lung CT scan was performed, showing pneumonia, bilateral pleural effusion, lymphadenopathy, as well as mild pericardial effusion (Fig. 1B). Ultrasound also suggested diffuse injury of both kidneys. Neck rashes worsened with de novo development of itchy rashes around the right quadrant abdomen, prothorax, arms, legs, and buttocks (Fig. 3A). Next-generation sequencing did not detect pathogens in pleural effusion, but suggested cytomegalovirus infection in peripheral blood and suspected infection of Torque teno virus, Epstein–Barr virus, and herpes simplex virus. Collectively, she simultaneously had recurrent fever, rash, lymphadenopathy, and organ dysfunction. According to the Registry of Severe Cutaneous Adverse Reactions to Drugs scoring criteria,^[7]^ she had a score of 5 points, including fever (0 points), lymphadenectasis (1 point), extension of rash beyond 50% of the body surface area (1 point), skin rash suggesting DRESS (1 point), heart, lung, kidney, liver, and other organ involvement (2 points). Thus, ‘probable DRESS’ was considered. After methylprednisolone and immunoglobulin, her temperature normalized on November 7, and rashes around the right quadrant abdomen and limbs gradually decreased (Fig. 3B). Lung CT scan was repeated on November 12, indicating the improvement of pneumonia and pleural effusion (Fig. 1C). Serum bilirubin and IgG levels also decreased (Fig. 4). Ganciclovir was stopped on November 15, and skin rashes completely disappeared on November 25. Methylprednisolone dosage was tapered during follow-up period (Fig. 3C). One month after discharge, her liver function and IgG levels remained normal. She was diagnosed with Sjogren syndrome at the Department of Rheumatology and Immunology, and took hydroxychloroquine. Glucocorticoids were gradually tapered.

## 3. Discussion

### 3.1. Diagnosis of DRESS and differential diagnosis:

Based on the diagnostic criteria for DRESS/drug-induced hypersensitivity syndrome in both Japan and Europe, increased eosinophils and leukocytes are considered 1 of the diagnostic criteria, but our patient had a decrease in white blood cells and eosinophils. This unexpected phenomenon might be attributed to the presence of AIH as well as Epstein–Barr virus reactivation and cytomegalovirus infection. Patients with autoimmune liver diseases may experience anemia, thrombocytopenia, and leukopenia. This is mainly due to the immune-mediated destruction of blood cells, as well as the development of portal hypertension and splenomegaly secondary to chronic liver diseases. This is also probably because chronic inflammation of the liver releases pro-inflammatory cytokines, such as tumor necrosis factor-α and IL-6, thereby inhibiting hematopoiesis in the bone marrow.^[8]^ On the other hand, patients with autoimmune diseases have IgE, IgG, and IgA antibodies, which can cause immune-mediated destruction of eosinophils in peripheral blood.^[9,10]^ It has been reported that eosinophils, lymphocytes, and basophils can be reduced during the acute phase of measles virus infection, and even temporarily lost.^[11]^ Besides, it has been demonstrated that eosinophils were absent in both peripheral blood and bone marrow in a 55-year-old male patient with a history of recurrent bacterial and viral infections, which might be related to impaired T-cell function.^[12]^ Taken together, our patient was not given a 1 point from increased eosinophils, so she was diagnosed with “probable DRESS.”

The nature of the rash in our patient should be differentiated from Stevens-Johnson syndrome, toxic epidermal necrolysis, acute generalized exanthematous pustulosis, and erythrodermic drug eruption, which often develop more than 48 hours and 3 weeks after exposure to sensitizing drugs. Their typical skin lesions are generalized maculopapules, which can be accompanied by urticaria-like and eczema-like lesions, significant skin edema, inflammation, and pustules. Our case had neither pustules nor skin mucosal erosion. Stevens-Johnson syndrome and toxic epidermal necrolysis are rarely accompanied with multiple system involvement, such as liver and lung, and the above diseases can be excluded by comprehensive clinical characteristics.^[13]^

The nature of the fever in our patient should be differentiated from infectious mononucleosis. Before admission, Epstein–Barr virus had been infected, but reactivated. After admission, cytomegalovirus infection was diagnosed. According to the Hoagland criteria, lymphocytes > 50% and 10% of atypical lymphocytes are required diagnostic criteria for infectious mononucleosis,^[14]^ but were not met in our patient.

### 3.2. Pathogenesis of DRESS:

Interaction of drugs, viral activation, and immune system is critical for the pathogenesis of DRESS.^[3,15]^ When the body is exposed to a specific drug, the immune balance of the body may be broken, and then, latent viruses are activated, including human herpes virus 6, 7, cytomegalovirus, and Epstein–Barr virus.^[16,17]^ At this stage, T cells, the core immune cells located in skin, liver, mucosa, and other tissues of the human body, undergo extensive proliferation, cross-recognition, and abnormal activation, which simultaneously target viruses, drugs, and autologous tissues. Consequently, this leads to rash, fever, lymphadenopathy, and multi-organ dysfunction, with symptoms continuing to deteriorate and recur even after drug discontinuation.^[18]^ On the other hand, viral infection can up-regulate the expression of antigens and co-stimulatory molecules, and enhance abnormal drug-immune receptor interaction through the p-i pathway, resulting in abnormal activation of human leukocyte antigen-restricted T cells and triggering cellular immune disorders.^[19]^

Our patient was treated with multiple drugs before admission and during hospitalization. Roxithromycin, a new generation of semisynthetic tetracyclic macrolide antibiotics, can cause drug-induced dermatitis, allergic purpura, drug eruption, and allergic reaction, which are generally immediate reactions and occur within 1 to 2 days since the use of medication. A prominent feature of DRESS is late-onset clinical symptoms following the exposure to a causative drug, in contrast to other common drug reactions, which typically manifest shortly after drug exposure. Because the onset of DRESS typically occurs 2 to 6 weeks after drug exposure, roxithromycin is not considered as the potential cause. In our case, the potentially causative drugs were ibuprofen, ketorolac, and indomethacin, all of which are NSAIDs, the most important drugs that induce DRESS. Um et al demonstrated that NSAIDs were the causative drugs in 13.2% of 38 patients with DRESS.^[20]^ Lee et al reported a 55-year-old female who developed DRESS following intramuscular administration of diclofenac.^[21]^ Kawakami et al also reported a 2-year-old Japanese boy with Kawasaki disease who developed DRESS following 1-month aspirin.^[22]^ Hypersensitivity reaction induced by NSAIDs can involve a variety of mechanisms with diverse clinical manifestations. The European Network for Drug Allergy interest group divides the hypersensitivity of NSAIDs into 2 categories.^[23]^ The first category is nonimmune-mediated hypersensitivity reaction, where the inhibition of cyclooxygenase-1 activates mast cells and eosinophils, and releases inflammatory mediators. The second category is immune-mediated hypersensitivity, namely allergic reaction.^[24]^ Specifically, in our case, ibuprofen, a nonselective cyclooxygenase inhibitor,^[25]^ was given 4 weeks before the development of DRESS, and ketorolac tromethamine and indomethacin suppository, of which both were nonselective cyclooxygenase inhibitors, were used about 1 week before the onset of the disease. Our patient might develop DRESS after the application of the 3 drugs. Marak et al reported that ketorolac tromethamine induced diffuse alveolar hemorrhage, but its pathogenesis and histopathological features are unclear.^[26]^ Notably, our patient had a large amount of bloody pleural effusion. Although sensitizing drugs have been discontinued, many herpes viruses can be reactivated in the event of a severe drug reaction, eventually leading to multiple organ failure after discontinuation. However, our patient was given antiviral drugs in time. Although renal color Doppler ultrasonography demonstrated diffuse renal injury, renal function parameters remained unremarkable in our patient. Additionally, pericardial effusion was identified in the absence of myocarditis.

### 3.3. Virus reactivation induces AIH:

Epstein–Barr virus and cytomegalovirus can induce the development of AIH.^[27,28]^ Ng et al reported that Epstein–Barr virus infection induced AIH in a 5-year-old Italian girl.^[29]^ Mouelhi et al reported that cytomegalovirus infection induced type 1 AIH in a 17-year-old female patient.^[30]^ Under the inducement of exogenous infection, such as Epstein–Barr virus, the immune homeostasis is disordered, and then, T and B lymphocytes initiate pathological autoimmune response simultaneously against hepatocyte-specific autoantigen. Persistent immune attack mediates progressive damage of hepatocytes, and eventually develops into AIH.^[31]^

## 4. Conclusion

If fever, rash, and organ dysfunction are observed, DRESS should be highly suspected in clinical practice. All potentially causative drugs should be comprehensively identified, including any drugs introduced within 8 weeks prior to the onset of DRESS. Subsequently, all known causative drugs and those with similar chemical structures should be avoided. Meanwhile, it is critical to monitor viral reactivation.^[32]^ Besides, the monitoring of autoimmune complications and viral reactivation is of vital importance. Systemic glucocorticoids combined with human immunoglobulin are still the main choices for the treatment of DRESS.^[7]^

## Author contributions

**Conceptualization:** Xingshun Qi.

**Data curation:** Shengwu Chuai, Yanhong Gao, Xuyong Lin, Xingshun Qi.

**Formal analysis:** Ran Wang, Ting Wang, Xingshun Qi.

**Funding acquisition:** Xingshun Qi.

**Investigation:** Shengwu Chuai, Yanhong Gao, Xingshun Qi.

**Methodology:** Shengwu Chuai, Yanhong Gao, Xingshun Qi.

**Project administration:** Shengwu Chuai, Ran Wang, Qianqian Li, Ting Wang, Xingshun Qi.

**Resources:** Xingshun Qi.

**Software:** Qianqian Li, Xingshun Qi.

**Supervision:** Xingshun Qi.

**Validation:** Xingshun Qi.

**Visualization:** Xingshun Qi.

**Writing** – **original draft:** Shengwu Chuai, Xingshun Qi.

**Writing** – **review & editing:** Shengwu Chuai, Xingshun Qi.
